# Muscle mass as a modifier of stress response in acute ischemic stroke patients

**DOI:** 10.1038/s41598-024-60829-6

**Published:** 2024-05-02

**Authors:** Ethem Murat Arsava, Levent Gungor, Hadiye Sirin, Mine Hayriye Sorgun, Ozlem Aykac, Hale Zeynep Batur Caglayan, Hasan Huseyin Kozak, Serefnur Ozturk, Mehmet Akif Topcuoglu, Muscle Assessment in Stroke Study (MASS) investigators, Erhan Akpinar, Mehmet Argın, Ustun Aydingoz, Ahmet Bugrul, Ezgi Sezer Eryildiz, Ayse Guler, Sevcihan Kesen, Bijen Nazliel, Atilla Ozcan Ozdemir, Sehriban Peynir, Ahmet Veysel Polat, Necdet Poyraz, Canan Togay Isikay, Caglar Uzun

**Affiliations:** 1 Department of Neurology, Faculty of Medicine, Hacettepe University, 06230 Altindag, Ankara Turkey; 2 Department of Neurology, Ondokuz Mayis University, Samsun, Turkey; 3 Department of Neurology, Ege University, Izmir, Turkey; 4 Department of Neurology, Ankara University, Ankara, Turkey; 5 Department of Neurology, Eskisehir Osmangazi University, Eskisehir, Turkey; 6 Department of Neurology, Gazi University, Ankara, Turkey; 7 Department of Neurology, Necmettin Erbakan University, Konya, Turkey; 8 Department of Neurology, Selcuk University, Konya, Turkey; 9 Department of Radiology, Hacettepe University, Ankara, Turkey; 10 Department of Radiology, Ege University, Izmir, Turkey; 11 Department of Radiology, Gazi University, Ankara, Turkey; 12 Department of Radiology, Ondokuz Mayis University, Samsun, Turkey; 13 Department of Radiology, Necmettin Erbakan University, Konya, Turkey; 14 Department of Radiology, Ankara University, Ankara, Turkey

**Keywords:** Cerebrovascular disorders, Stroke, Biomarkers

## Abstract

Stroke triggers a systemic inflammatory response over the ensuing days after the cerebral insult. The age and comorbidities of the stroke population make them a vulnerable population for low muscle mass and sarcopenia, the latter being another clinical condition that is closely associated with inflammation, as shown by increased levels of pro-inflammatory biomarkers, including neutrophil-to-lymphocyte ratio (NLR). In this study, we evaluated the relationship between post-stroke NLR changes and muscle mass in a prospective cohort of acute ischemic stroke patients (n = 102) enrolled in the Muscle Assessment in Stroke Study Turkey (MASS-TR). Admission lumbar computed tomography images were used to determine the cross-sectional muscle area of skeletal muscles at L3 vertebra level and calculate the skeletal muscle index (SMI). The median (IQR) SMI was 44.7 (39.1–52.5) cm^2^/m^2^, and the NLR at admission and follow-up were 4.2 (3.0–10.5) and 9.4 (5.7–16.2), respectively. While there was no relationship between SMI and admission NLR, a significant inverse correlation was observed between SMI and follow-up NLR (r =  − 0.26; *P* = 0.007). Lower SMI remained significantly associated (*P* = 0.036) with higher follow-up NLR levels in multivariate analysis. Our findings highlight the importance of muscle mass as a novel factor related to the level of post-stroke stress response.

## Introduction

Ischemic stroke, similar to many other severe illnesses that necessitate critical care admission (such as sepsis, trauma, burns, major surgery, etc.), is well known to trigger an acute stress response. Although the intensity of this response is closely related with the severity of the clinical deficit or the size of the ischemic tissue, it still has independent prognostic implications in terms of early or long-term outcomes^1–4^. Among various biomarkers of this acute stress response, the neutrophil-to-lymphocyte ratio (NLR) is a simple, but readily available metric and has been extensively studied in the acute stroke population. The elevated NLR following stroke reflects neutrophil demargination and lymphocyte apoptosis as a result of sympathetic pathway and hypothalamus–pituitary–adrenal axis activation, and is also considered a part of the pro-inflammatory status accompanying the initial days of stroke^5,6^. Elevated NLR at admission or follow-up was demonstrated to be a predictor of stroke-associated pneumonia, hemorrhagic complications, unfavorable functional outcome, and death^7–13^.

Stroke, typically a disease of the elderly population, is frequently accompanied by some degree of muscle mass loss or sarcopenia even at the time of hospital admission, which increases over the course of the disease and contributes to an unfavorable outcome^14–17^. One key aspect in the pathophysiology of primary or secondary sarcopenia is its intricate relationship with systemic inflammation. On one hand, there is evidence of a chronic inflammatory state and elevated inflammatory cytokine levels in sarcopenic patients^18–20^. In this regard, NLR was found to be elevated in patients with primary sarcopenia^21,22^ and cancer with an accompanying loss in muscle mass^23,24^. On the other hand patients with low skeletal muscle mass reserve are prone to develop an increased inflammatory response in the body, as primarily shown by studies performed in sepsis and perioperative patients^25–27^.

Evaluation of the skeletal muscle area at the level of L3 vertebra level by magnetic resonance imaging or computed tomography is considered to accurately reflect the muscle mass content of the whole body and can be used as a supportive tool in diagnosing sarcopenia^28,29^. The primary goal of the current study was to assess whether the observations on muscle mass status and inflammatory response could be applied to the acute ischemic stroke population, specifically by examining the relationship between admission skeletal muscle index (SMI) and early course of NLR in a consecutive series of major ischemic stroke patients.

## Results

### Study population

A total of 124 patients were enrolled in the Muscle Assessment in Stroke Study. One patient withdrew consent after enrollment, and another was excluded due to a new diagnosis of cancer. Among the remaining patients, reliable determination of CSMA at the level of L3 vertebra was not possible in 19 patients due to imaging artifacts and technical issues related to image acquisition. Neutrophil and lymphocyte counts were not available at the prespecified time points in another patient. Therefore, the current study population was comprised of 102 patients.

### Baseline characteristics of the study population

Table 1 summarizes the baseline characteristics of the study population. The median (interquartile range, IQR) cross-sectional muscle area (CSMA) of the muscles at the L3-vertebra level was 114.4 (96.9–114.0) cm^2^, and the SMI was 44.7 (39.1–52.5) cm^2^/m^2^. Male patients had higher SMI [50.8 (44.3–56.2) vs. 40.4 (36.3–45.4) cm^2^/m^2^; *P* < 0.001] and CSMA’s [142.1 (126.6–159.6) vs. 98.2 (88.3–111.8) cm^2^; *P* < 0.001] in comparison to females. Expectedly, older age was negatively correlated both with SMI (r = − 0.43; *P* < 0.001) and CSMA (r = − 0.51; *P* < 0.001). Admission NLR obtained after a median (IQR) of 4.2 (2.0–7.6) hours after stroke onset was 4.2 (3.0–10.5). This value increased to 9.4 (5.7–16.4) in the follow-up blood samples collected after a median duration of 36.2 (24.5–43.4) hours following symptom onset, yielding a median follow-up-to-admission NLR ratio of 1.6 (0.9–3.2).

**Table 1:** Baseline characteristics of the study population.

	Study population (n = 102)
Age, years (median, IQR)	74 (61–79)
Female sex (n, %)	54 (53%)
Risk factors (n, %)	
Hypertension	74 (73%)
Diabetes mellitus	38 (37%)
Hyperlipidemia	23 (23%)
Coronary artery disease	39 (38%)
Admission NIHSS score (median, IQR)	17 (13–22)
Admission body temperature, °C (median, IQR)	36.9 (36.3–37.4)
Body height, cm (median, IQR)	160 (152–170)
Body weight, kg (median, IQR)	73 (65–80)
Body mass index, kg/m^2^ (median, IQR)	27.0 (24.7–30.8)
Total CSMA of muscles at L3-vertebra level, cm^2^ (median, IQR)	114.4 (96.9–114.0)
Skeletal muscle index, cm^2^/m^2^ (median, IQR)	44.7 (39.1–52.5)
Admission neutrophil count, × 10^3^/mm^3^ (median, IQR)	6.9 (5.3–9.7)
Admission lymphocyte count, × 10^3^/mm^3^ (median, IQR)	1.4 (0.9–2.1)
Admission NLR (median, IQR)	4.2 (3.0–10.5)
Follow-up neutrophil count, × 10^3^/mm^3^ (median, IQR)	9.8 (7.2–12.6)
Follow-up lymphocyte count, × 10^3^/mm^3^ (median, IQR)	1.0 (0.7–1.5)
Follow-up NLR (median, IQR)	9.4 (5.7–16.4)

Numerical variables are expressed as median (interquartile range).

*CSMA* cross-sectional muscle area, *IQR* interquartile range, *NIHSS* National Institutes of Health Stroke Scale, *NLR* neutrophil lymphocyte ratio.

### Relationship between NLR and skeletal muscle indices

**Figure 1:**
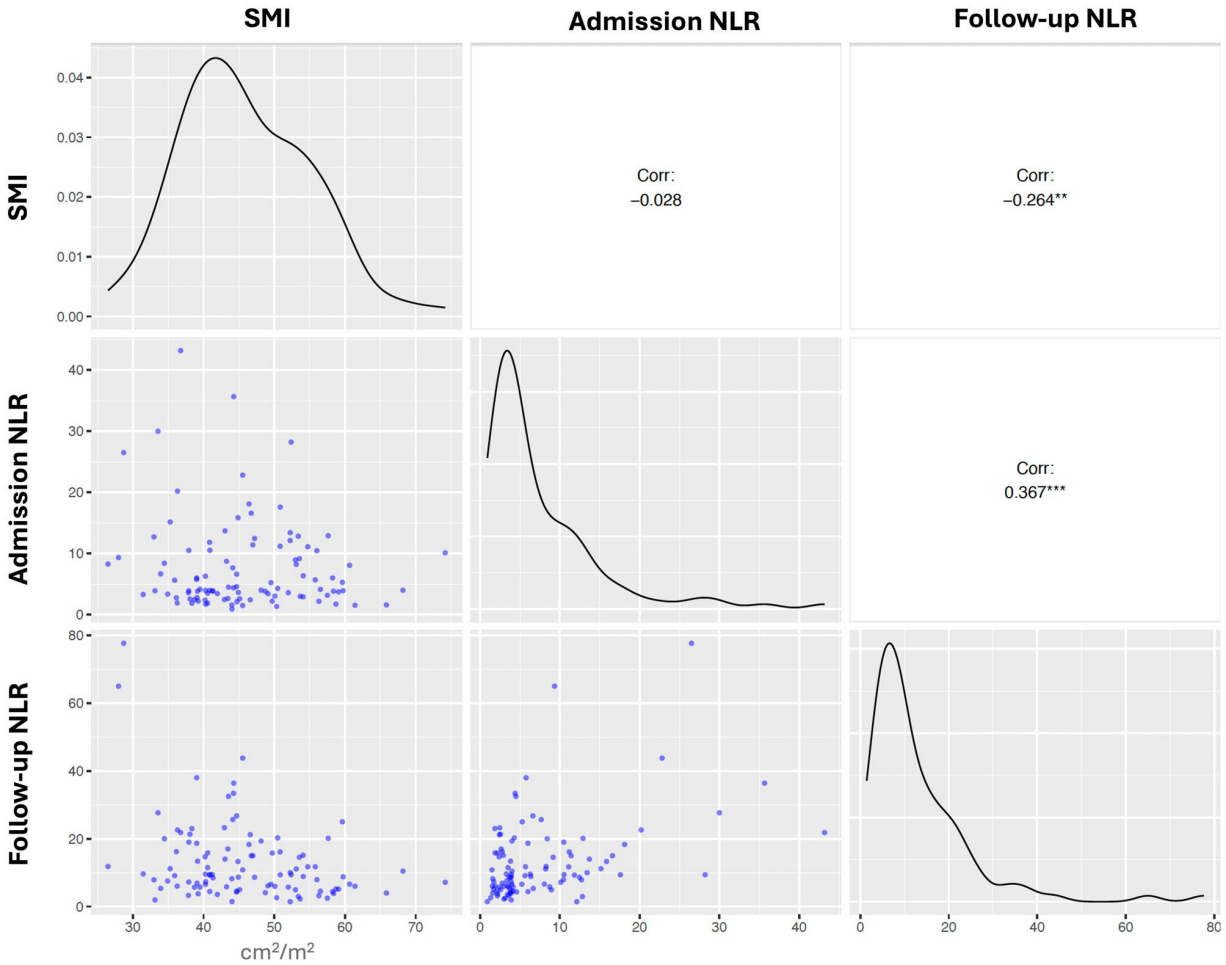
The correlogram of the study variables. The diagonal panels summarize the distribution of each variable, the lower left-hand panels demonstrate the relationship between variables with scatterplots, and the upper right-hand panels show the Spearman correlation coefficients. **p < 0.01; ***p < 0.001.

The relationship of admission and follow-up (measured 24 to 48 h after stroke onset) NLR levels with patient characteristics and skeletal muscle indices are summarized in Table 2 and Fig. 1. Admission NLR was not significantly related to any of the clinical or imaging variables, except for the time to blood sample collection. On the other hand, older age (r = 0.28; *P* = 0.004), higher stroke severity (r = 0.27; *P* = 0.006) and lower skeletal muscle cross-sectional area (r = − 0.21; *P* = 0.033) or SMI (r = − 0.26; *P* = 0.007) were associated with a higher NLR on follow-up.

**Table 2:** The relationship of admission and follow-up NLR levels with clinical features and skeletal muscle indices.

	Admission NLR	Follow-up NLR
Age	r = 0.11; *P* = 0.279	**r = 0.28; *P* = 0.004**
Female sex	*P* = 0.909	*P* = 0.311
Risk factors		
Hypertension	*P* = 0.564	*P* = 0.094
Diabetes mellitus	*P* = 0.271	*P* = 0.491
Hyperlipidemia	*P* = 0.278	*P* = 0.648
Coronary artery disease	*P* = 0.284	*P* = 0.531
Admission NIHSS score	r = 0.12; *P* = 0.215	**r = 0.27; *P* = 0.006**
Admission body temperature	r = 0.07;* P* = 0.511	r = − 0.11;* P* = 0.300
Body mass index	r = − 0.07; *P* = 0.497	r = − 0.09; *P* = 0.398
Total CSMA of muscles at L3-vertebra level	r = 0.01; *P* = 0.938	**r = − 0.21; *P* = 0.033**
Skeletal muscle index	r = **− **0.03; *P* = 0.784	**r = − 0.26; *P* = 0.007**
Time to admission blood sampling	**r = 0.38; *P* < 0.001**	r = **− **0.09; *P* = 0.366
Time to follow-up blood sampling	–	r = **− **0.17; *P* = 0.092

Bivariate comparisons were performed by Mann–Whitney U test for categoric/continuous variables and by Spearman’s correlation test for continuous/continuous variables.

*CSMA* cross-sectional muscle area, *NIHSS* National Institutes of Health Stroke Scale, *NLR* neutrophil lymphocyte ratio

Siignificant values are in bold.

The results of the multivariate analyses are summarized in Table 3. Lower SMI was significantly associated [β (SE) = − 0.020 (0.010), *P* = 0.036] with higher follow-up NLR levels when adjusted for age, sex, stroke severity, and time to blood sampling. Other variables significantly associated with follow-up NLR included age [β (SE) = 0.013 (0.006), *P* = 0.039] and stroke severity [β (SE) = 0.037 (0.014), *P* = 0.007]. The association between SMI and change in NLR levels persisted when the analyses were repeated by constructing the model with the ratio of follow-up to admission NLR as the dependent variable (Supplemental Table S1).

**Table 3:** Multivariate predictors of NLR on follow-up.

	β (SE)	*P*
Age	0.013 (0.006)	**0.039**
Female Sex	− 0.130 (0.164)	0.431
Hypertension	0.075 (0.173)	0.665
Admission NIHSS score	0.037 (0.014)	**0.007**
Time to follow-up blood sampling	− 0.007 (0.005)	0.129
Skeletal muscle index	− 0.020 (0.010)	**0.036**

The independent variables in the model included those variables with a *P* value of ≤ 0.100 in the bivariate analysis stage, together with the additional variable sex due to its close relationship muscle mass.

*NIHSS* National Institutes of Health Stroke Scale, *NLR* neutrophil lymphocyte ratio.

Siignificant values are in bold.

## Discussion

Our results highlight elevated NLR response in the early post-acute stroke period among patients with a lower SMI. This relationship persisted when the analyses were adjusted for other important confounders associated with NLR dynamics in stroke patients, including age and stroke severity. Overall, these findings highlight the importance of muscle mass as a novel factor related to level of post-stroke stress response.

The post-stroke setting is intricately related to a multitude of changes in the systemic inflammation pathways. The hyperacute change in this multi-phasic cascade of events is a drift towards a proinflammatory status, followed sequentially over days and weeks toward immunodepression with a switch back towards immune activation in the chronic phase^6,30^. NLR elevation could be considered among the various indices showing the presence of a proinflammatory milieu in the acute phase of stroke, as its levels have been shown to elevate even in the absence of post-stroke infections as part of the post-stroke systemic stress response^8–13,31^. Concordantly, a more severe stroke, determined either by higher admission NIHSS scores or larger lesion volumes, is closely related to higher post-stroke NLR levels^7,10,11,13,31^. Nevertheless, even after controlling for the severity of the ischemic insult, the main driver of the post-stroke inflammatory response, there is still a significant amount of inter-individual variation among stroke patients in terms of the presence and extent of this phenomenon^2^. Aging, sex, and strategic locations of cerebral infarcts have been highlighted as factors that contribute to this variability^32–34^. Our findings now add the muscle mass reserve of the individual to this list. Considering the neutral association at the time of admission, which was quite early after the cerebral insult in our cohort (around four hours between symptom onset and initial blood sampling), it is plausible to speculate that the interplay between reduced muscle mass and NLR increase becomes apparent after a certain time lag, possibly as a reflection of the pro-inflammatory status intensifying over time after stroke.

Muscle tissue has a bidirectional relationship with the immune system. On one hand, the presence of systemic inflammation adversely affects muscle health, while at the same time, muscle tissue acts as a regulator of the immune system^35^. The disruption of the normal muscle physiology, as in the case of sarcopenia, has been shown to lead to abnormal myokine signaling culminating in a proinflammatory environment characterized by impaired IL-6 signaling, increased TNF-alpha production and decreased IL-1ra and IL-10 levels^35^. Although this literature primarily focuses on chronic inflammation and muscle health, the influence of muscle mass on acute stress responses has been an active area of research in the field of surgery. The presence of sarcopenia or low skeletal muscle mass content was shown to be associated with a higher postoperative inflammatory response and thereby was considered to underlie higher postoperative morbidity and mortality in these patients^27,36^. The acute stroke setting, can also be considered to simulate a similar scenario, and as shown by our results is also characterized by a more prominent stress response among patients with low muscle mass.

The use of a prospectively collected and well-studied patient population and the measurement of muscle mass by a highly reliable radiological tool are the major strengths of the study. On the other hand, a number of limitations of the current study should also be taken into account. First of all, the acute stress response following stroke was evaluated only via NLR, which could be considered a crude and non-specific marker of inflammation. Although post-stroke infections generally contribute to NLR elevations after 48 h following the stroke onset, it is not entirely possible to rule out their contribution to the follow-up NLR elevations in our cohort^13^. Another major limitation was the absence of information on sarcopenia, as such a formal evaluation was not present in any of our patients prior to the stroke and was not possible after their admission. Therefore, our findings are only applicable to muscle mass, and could not be extrapolated to other aspects of sarcopenia which include muscle strength and physical performance. Additionally, due to the relatively low sample size of the study population, we were not able to tease out the independent roles of low muscle mass and post-stroke NLR elevations for unfavorable stroke outcomes. The lack of information on patients about the presence of a pre-stroke rheumatologic disease or the use of any anti-inflammatory medications, together with the absence of data related to early infections until follow-up blood sampling are other important limitations of the study. Finally, the fact that our study cohort was predominantly composed of patients with moderate to severe impairments limits the generalizability of our findings to the overall stroke population.

In conclusion, our results highlight the acute ischemic stroke setting, as an additional arena where muscle health and systemic inflammatory response closely interact with each other. The clinical significance of our observations necessitates further studies. Previous research has demonstrated that a reduced muscle mass and an elevated inflammatory milieu both have a negative impact on stroke outcomes. A possible association between these two negative prognostic factors, as highlighted by our results, deserves further attention for their possible additive or synergistic effects. Future studies with larger number of patients that make use of various markers related to the functions of the immune system, neuroimaging analyses for potential confounding related to specific infarct locations associated with systemic responses, and tools to systemically assess sarcopenia are needed to clarify the mechanisms of this interplay, as well as its clinical implications from the perspective of prognosis and therapeutic opportunities.

## Methods

This study was a secondary analysis of the data obtained as part of the multi-center, prospective and observational Muscle Assessment in Stroke Study (MASS) (ClinicalTrials.gov identifier: NCT03825419), the primary results of which were published recently^37^. Briefly, the study population was comprised of adult ischemic stroke patients admitted to neuro-intensive care units within 24 h of symptom onset. Inclusion criteria were the presence of an admission National Institute of Health Stroke Scale (NIHSS) score ≥ 8 and the presence of dysphagia necessitating enteral tube feeding. Patients with pregnancy, co-morbid diseases leading to chronic immobilization prior to stroke, active cancer, and chronic liver or renal failure were excluded from the study. The study protocol was approved by the Institutional Ethics Committee of the coordinating center (Ondokuz Mayis University, Samsun, Turkiye). All research-related procedures have been performed in accordance with the Declaration of Helsinki, and written informed consent was obtained from all participants and/or their legal guardians.

The following variables were assessed for the purposes of the current study: age, gender, vascular risk factors (hypertension, diabetes mellitus, coronary artery disease, hyperlipidemia), and stroke etiology. All patients were weighed at the time of admission by the weighing scales of the intensive care unit beds. This information, together with body height measured by a flexible measuring tape, was used to calculate body mass index (BMI). The absolute neutrophil and lymphocyte levels obtained at admission and 24 to 48 h after stroke onset were used to determine NLR at both time points (admission and follow-up NLR, respectively).

The primary aim of MASS was the determination of changes in cross-sectional muscle areas (CSMA) within 14 days of stroke and factors contributing to these changes. Therefore, all patients were imaged by computed tomography (CT) on days 1 and 14 after admission for the determination of CSMA in the mid-arm, mid-thigh and L3 vertebra levels. The details on image acquisition and analysis protocol were presented in the primary publication^37^. Briefly, imaging was performed by multi-detector CT scanners (matrix 512 × 512, tube voltage 120 kVP, slice thickness 5 mm) at the three abovementioned anatomic sites. For the purposes of the current study, only images obtained at the third vertebral level were used. Semi-automated segmentation was performed on the anonymized Digital Imaging and Communications in Medicine (DICOM) images using a threshold set between − 29 and + 150 HU to create muscle tissue masks for the skeletal muscles. The skeletal muscle index (SMI) was calculated by dividing the total CSMA of all the skeletal muscles at the L3 vertebra level to the square of body height^38^. All imaging analyses were performed while blinded to the clinical data using a publicly available image processing software (ImageJ version 1.53e, Bethesda, Maryland, USA)^39^.

### Statistical analysis

Categorical variables are expressed as n (%) and numerical variables as median (interquartile range, IQR) or mean ± standard deviation (SD). Normality was assessed by Kolmogorov–Smirnov test. Bivariate analyses were performed by chi-square, Spearman correlation and Mann–Whitney U tests, accordingly. Linear regression models were used for multivariate analysis. The dependent variables (follow-up NLR) were log-normalized in order to comply with the assumption of normality. Independent variables introduced into the model were selected among those with a p-value of ≤ 0.10 at the bivariate analysis stage. The statistical analyses were performed by SPSS Statistics version 22.0 (IBM Corp., Armonk, NY, USA) and R (R Foundation for Statistical Computing, Vienna, Austria)^40^. A *P* value of < 0.05 was considered statistically significant.

### Supplementary Information

Supplementary Table 1.

## Data Availability

The datasets generated and/or analysed during the current study are not publicly available as the data ownership belongs to all MASS-TR investigators who are still working on the data for further publications. However, the dataset for the current study is available from the corresponding author upon reasonable request.
