# Supplementary material for: Muscle mass as a modifier of stress response in acute ischemic stroke patients
Source: Sci Rep. 2024 May 2;14:10088. doi: 10.1038/s41598-024-60829-6 (PMC11066052; doi:10.1038/s41598-024-60829-6)
Supplement: Supplementary file 1 — Supplementary Table 1. [file 41598_2024_60829_MOESM1_ESM.docx]

**Supplemental Table 1:** Multivariate predictors of follow-up to admission NLR ratio

|  | **β (SE)** | ***P*** |
| --- | --- | --- |
| Age | 0.005 (0.007) | 0.448 |
| Female Sex | -0.093 (0.186) | 0.619 |
| Hypertension | -0.027 (0.194) | 0.888 |
| Admission NIHSS score | 0.011 (0.015) | 0.481 |
| Time to admission blood sampling | -0.049 (0.018) | **0.007** |
| Time to follow-up blood sampling | -0.006 (0.007) | 0.395 |
| Skeletal muscle index | -0.022 (0.011) | **0.043** |

CSMA: cross-sectional muscle area; NIHSS: National Institutes of Health Stroke Scale; NLR: Neutrophil lymphocyte ratio.
